# Global pharmacovigilance overview of adverse drug reactions associated with propofol: A descriptive pharmacovigilance study using WHO-VigiAccess data

**DOI:** 10.1097/MD.0000000000050189

**Published:** 2026-08-14

**Authors:** Nasser M. Alorfi, Ammar Aldabbagh, Mansour M. Alourfi, Waleed Saleh Alghamdi, Sarah S. Al Ghamdi, Ahmed Abdullah Niyazi, Nasser M. Aldekhail, Mustfa Faisal Alkhanani, Nihal Abdalla Ibrahim, Abdulrhman M. Alansari, Tariq Omar Ali, Faris A. Alsolami, Samah H.O. Zarroug, Hana K. Abdalla

**Affiliations:** a Pharmacology and Toxicology Department, College of Pharmacy, Umm Al‐Qura University, Makkah, Saudi Arabia; b Gastroenterology Department, Dr. Soliman Fakeeh Hospital, Fakeeh Care Group, Jeddah, Saudi Arabia; c Department of Medicine, Faculty of Medicine, King Abdulaziz University, Jeddah, Saudi Arabia; d Division of Gastroenterology, East Jeddah Hospital, Jeddah, Saudi Arabia; e Department of Pharmacy, College of Pharmacy, Nursing and Medical Sciences, Riyadh Elm University, Riyadh, Saudi Arabia; f Biology Department, College of Sciences, University of Hafr Al Batin, Hafr Al Batin, Saudi Arabia; g College of Pharmacy, Ajman University, Ajman, United Arab Emirates; h Center of Medical and Bio‐Allied Health Sciences Research, Ajman University, Ajman, United Arab Emirates; i Pharmaceutical Care Services, King Salman Medical City, Madinah Health Cluster, Madinah, Saudi Arabia; j Pharmaceutical Care Services, Ohoud, Madinah Health Cluster, Madinah, Saudi Arabia; k Kholais General Hospital, Makkah Cluster, Ministry of Health, Saudi Arabia; i Department of Pharmacology and Therapeutics, College of Medicine, Alfaisal University, Riyadh, Saudi Arabia; m Department of Microbiology and Immunology, College of Medicine, Alfaisal University, Riyadh, Saudi Arabia.

**Keywords:** adverse drug reactions, anesthesia, pharmacovigilance, propofol, safety profile, WHO-VigiAccess

## Abstract

Propofol is an intravenous anesthetic widely used for induction and maintenance of anesthesia and sedation. Despite its well-established efficacy and routine clinical use, multiple reports have described adverse reactions ranging from mild events to life-threatening complications. Pharmacovigilance databases provide valuable insight into such real-world experiences. This study provides an updated global overview of adverse drug reactions (ADRs) associated with propofol using data from the World Health Organization (WHO) VigiAccess platform. A retrospective descriptive study was performed using publicly available data from the WHO-VigiAccess database, retrieved on October 26,2025, covering all spontaneous reports available from the inception of WHO pharmacovigilance reporting through the search date. ADRs were summarized according to the Medical Dictionary for Regulatory Activities (MedDRA) System Organ Class and analyzed by geographical region, sex, and age group. No individual case review, disproportionality analysis, or causality assessment was performed. VigiAccess listed 45 354 ADR reports for propofol. Of the 45,354 ADR reports, skin and subcutaneous tissue disorders (12%), vascular disorders (12%), general disorders and administration site conditions (11%), nervous system disorders (9%), respiratory disorders (9%), immune disorders (8%), and cardiac disorders (8%) were most frequently reported. Europe and the Americas accounted for the largest proportion of reports. Male patients represented a slightly higher proportion than females, and adults aged 45 to 64 years constituted the most frequently reported age group. Temporal analysis revealed a continuous increase in reporting over the past 2 decades. This descriptive pharmacovigilance overview highlights the broad spectrum of spontaneously reported ADRs associated with propofol and emphasizes the importance of continued pharmacovigilance and post-marketing safety monitoring.

## 1. Introduction

Propofol, a short-acting alkylphenol derivative, is one of the most widely used intravenous (IV) anesthetic agents worldwide.^[1,2]^ It facilitates rapid induction and recovery from anesthesia due to its favorable pharmacokinetic properties.^[3-5]^ Propofol is primarily used as an IV anesthetic agent for the induction and maintenance of general anesthesia, sedation during surgical or diagnostic procedures, and sedation of mechanically ventilated patients in intensive care settings.^[6-8]^ The clinical dosing recommendations for propofol in anesthesia and procedural sedation are presented in Table 1, adapted from the British National Formulary, 90th Edition (2025).

**Table 1:** Clinical indications and recommended doses of propofol (BNF 2025).

Indication	Formulation/concentration	Route of administration	Population/age group	Recommended dose
Induction of anesthesia	0.5% or 1% injection	Slow IV injection or IV infusion	Adults 18–54 yr	1.5–2.5 mg/kg, administered at 20–40 mg every 10 s until clinical response. For debilitated patients, use elderly dosing
			Adults ≥ 55 yr	1–1.5 mg/kg, administered at 20 mg every 10 s until response
Induction of anesthesia	2% injection	IV infusion	Adults 18–54 yr	1.5–2.5 mg/kg, at 20–40 mg every 10 seconds until response. Use lower dose for debilitated patients
			Adults ≥ 55 yr	1–1.5 mg/kg, at 20 mg every 10 seconds until response
Maintenance of anesthesia	1% injection	IV infusion (initially), or slow IV injection	Adults	4–12 mg/kg/h (infusion); or 25–50 mg slow IV injection, repeated according to response. Use elderly dose for debilitated patients
			Elderly	3–6 mg/kg/h (infusion); or 25–50 mg slow IV injection, repeated as required
Maintenance of anesthesia	2% injection	IV infusion	Adults	4–12 mg/kg/h. Use elderly dose for debilitated patients
			Elderly	3–6 mg/kg/h
Sedation of ventilated patients in intensive care	1% or 2% injection	Continuous IV infusion	Adults	0.3–4 mg/kg/h, adjusted according to clinical response
Induction of sedation for surgical or diagnostic procedures	0.5% or 1% injection	Slow IV injection	Adults	0.5–1 mg/kg over 1–5 minutes; dose and rate adjusted according to desired sedation level
Maintenance of sedation for surgical or diagnostic procedures	0.5% injection	IV infusion (initially), followed by slow IV injection if required	Adults	1.5–4.5 mg/kg/h, adjusted according to response. For rapid sedation, additional 10–20 mg slow IV injection may be given. Lower initial dose recommended for patients > 55 yr or debilitated
Maintenance of sedation for surgical or diagnostic procedures	1% injection	IV infusion (initially), followed by slow IV injection if required	Adults	1.5–4.5 mg/kg/h; ma

According to the British National Formulary (2025), propofol should be administered only by trained personnel experienced in anesthesia and airway management, and only in facilities equipped with appropriate resuscitation equipment. Caution is required in patients with acute circulatory failure, cardiac impairment, cardiovascular disease, hypotension, hypovolemia, fixed cardiac output, raised intracranial pressure, respiratory impairment, epilepsy, or in the elderly and debilitated.^[9-13]^ Use with care in hepatic or renal impairment and monitor blood lipid concentrations in patients receiving prolonged infusions exceeding 3 days due to the risk of fat overload.

Propofol is associated with several dose-dependent adverse effects that warrant careful monitoring during administration. Common or very common adverse effects include apnea, arrhythmias, headache, hypotension, local pain on injection, nausea, and vomiting, while uncommon events such as thrombosis have also been reported.^[14-18]^ Rare or very rare reactions include epileptiform seizures, pancreatitis, pulmonary edema, urine discoloration, and soft tissue necrosis.^[19-22]^ Adverse effects with unknown frequency include dyskinesia, euphoric mood, drug use disorders, metabolic acidosis, respiratory depression, heart failure, rhabdomyolysis, renal failure, hepatomegaly, hyperkaliemia, and hyperlipidemia.^[23-27]^ Of particular clinical concern is propofol infusion syndrome, a potentially fatal complication associated with prolonged administration at doses exceeding 4 mg/kg/h, presenting with metabolic acidosis, arrhythmias, cardiac failure, rhabdomyolysis, and multiorgan dysfunction.^[28-31]^ Bradycardia may occur and can be treated with IV antimuscarinic agents, whereas injection-site pain may be mitigated with lidocaine pre-administration.^[32-35]^ Propofol may cause neonatal respiratory depression if used during delivery, and breastfeeding can resume once the mother has fully recovered from anesthesia^[36,37]^

Although controlled clinical trials have characterized many of propofol’s adverse reactions, spontaneous pharmacovigilance reporting systems provide complementary evidence derived from real-world contexts. These reports capture post-marketing data beyond trial conditions, allowing early detection of rare, delayed, or population-specific events. The World Health Organization (WHO) Programme for International Drug Monitoring, coordinated by the Uppsala Monitoring Centre, maintains the global VigiBase repository, which underpins the publicly accessible VigiAccess portal. Analyses of VigiAccess data have contributed to identifying novel safety patterns across numerous therapeutic classes.

Despite decades of clinical use, comprehensive global summaries of spontaneously reported propofol-associated adverse drug reactions (ADRs) remain limited. Because propofol is administered across diverse clinical settings worldwide, international pharmacovigilance data provide valuable insight into the spectrum of reported adverse events that may not be fully captured during premarketing clinical trials. Although spontaneous reporting systems cannot estimate incidence or establish causality, they are essential for identifying reporting patterns, generating safety hypotheses, and informing ongoing pharmacovigilance activities.

Given the high global usage of propofol and ongoing reports of potentially serious ADRs, this study aims to summarize the current international pharmacovigilance profile of propofol using WHO-VigiAccess data updated to October 26,2025. This analysis focuses on organ-system involvement, patient demographics, and reporting trends to support clinicians and safety regulators in monitoring its global safety landscape.

## 2. Materials and methods

### 2.1. Study design

A descriptive, retrospective study design was used to characterize aggregated ADR data related to propofol.

### 2.2. Data source

Data were obtained from the WHO-VigiAccess platform (https://vigiaccess.org/), developed by the Uppsala Monitoring Centre on behalf of the WHO Programme for International Drug Monitoring. The dataset was accessed on October 26,2025, corresponding to the most recent database update at that time.

### 2.3. Data extraction

The following variables were retrieved:

Total number of ADRs attributed to propofol; Distribution of ADRs by MedDRA System Organ Class (SOC); Geographic origin (Africa, Americas, Asia, Europe, Oceania); Sex and age group distribution; Year-by-year reporting trends. ADRs were categorized according to the Medical Dictionary for Regulatory Activities (MedDRA) System Organ Class (SOC), which represents the highest level of the MedDRA hierarchical terminology. SOC categorization enables standardized international comparison of ADR reporting across organ systems.

### 2.4. Data analysis

Data were analyzed descriptively, with absolute counts and percentages calculated for each SOC and demographic variable. Because the public WHO-VigiAccess platform provides only aggregated spontaneous reporting data, descriptive statistics (frequencies and percentages) were considered the most appropriate analytical approach. No inferential statistics, disproportionality analyses, or causality assessments were performed.

### 2.5. Ethical considerations

No ethics approval was required, as the study relied solely on de-identified, publicly available data from the WHO database.

### 2.6. Characteristics and limitations of the WHO-VigiAccess database

WHO-VigiAccess provides aggregated spontaneous ADR reports submitted to the WHO Programme for International Drug Monitoring. Because only summarized data are publicly available, individual case-level information, concomitant medications, dosage, treatment duration, patient comorbidities, and clinical outcomes cannot be examined. Furthermore, VigiAccess does not permit disproportionality analyses (e.g., reporting odds ratios, proportional reporting ratios, Bayesian analyses) or comparisons with other drugs because individual case reports and exposure denominators are unavailable. Consequently, this study was designed as a descriptive pharmacovigilance overview rather than a signal detection analysis.

## 3. Results

### 3.1. Overall reporting

As of October 26,2025, the WHO-VigiAccess platform listed 45 354 individual case reports associated with propofol.

### 3.2. Distribution by system organ class

As shown in Table 2, the leading SOC categories were skin and subcutaneous tissue disorders (12%), vascular disorders (12%), and general disorders and administration site conditions (11%). Other major groups included nervous system disorders (9%), respiratory disorders (9%), immune system disorders (8%), and cardiac disorders (8%).

**Table 2:** Adverse drug reaction distribution by MedDRA System Organ Class (SOC).

System Organ Class	ADRs (n)	% of total
Skin and subcutaneous tissue disorders	8732	12
Vascular disorders	8457	12
General disorders and administration site conditions	8080	11
Nervous system disorders	6413	9
Respiratory, thoracic and mediastinal disorders	6059	9
Immune system disorders	5692	8
Cardiac disorders	5668	8
Investigations	4118	6
Injury, poisoning and procedural complications	3573	5
Gastrointestinal disorders	3195	5
Metabolism and nutrition disorders	2042	3
Psychiatric disorders	1782	3
Musculoskeletal and connective tissue disorders	1519	2
Infections and infestations	918	1
Renal and urinary disorders	908	1
Others (each <1%)	1090	2

ADR = adverse drug reaction, MedDRA – Medical Dictionary for Regulatory Activities, SOC = system organ class.

### 3.3. Demographic characteristics

The public WHO-VigiAccess platform provides aggregated demographic information according to patient sex, age group, and geographical region. Male patients accounted for a slightly higher proportion of reports than female patients, while the highest reporting frequency was observed among adults aged 45 to 64 years, followed by those aged 18 to 44 years. Reports involving pediatric patients were comparatively infrequent. Geographically, the largest number of reports originated from the Americas and Asia, followed by Europe, reflecting differences in reporting activity among participating regions. Detailed demographic characteristics are summarized in Table 3.

**Table 3:** Demographic summary of propofol ADR reports.

Variable	Categories reported	Predominant category*	Data source
Sex	Male, female, unknown	Male	WHO-VigiAccess patient sex distribution
Age group	0–27 d; 1–23 mo; 2–11 yr; 12–17 yr; 18–44 yr; 45–64 yr; 65–74 yr; ≥75 yr; unknown	45–64 yr	WHO-VigiAccess age-group distribution
Geographic region	Africa; Americas; Asia; Europe; Oceania	Americas and Asia (followed by Europe)†	WHO-VigiAccess geographical distribution

ADR = adverse drug reaction, WHO = World Health Organization.

### 3.4. Temporal reporting trends

The number of propofol-associated ADR reports increased progressively over time (Table 4). During the early post-marketing period (1985–1989), only 431 reports were recorded, followed by gradual increases during 1990 to 1994 (n = 2028), 1995 to 1999 (n = 1666), 2000 to 2004 (n = 1188), and 2005 to 2009 (n = 2240). Reporting increased more substantially during 2010 to 2014, with 5878 ADR reports, and continued to rise during 2015 to 2019 (n = 9848). The highest reporting volume was observed during 2020 to 2024, accounting for 17,328 reports, while 4747 reports were recorded in 2025 up to the study search date. Overall, these findings demonstrate a sustained increase in spontaneous reporting of propofol-associated ADRs over the study period, likely reflecting expanded global pharmacovigilance activities, increased clinical utilization, and improved awareness and reporting of ADRs.

**Table 4:** Five-year reporting trend of adverse drug reaction (ADR) reports associated with propofol in the WHO-VigiAccess database (1985–2025).

Reporting period	ADR reports, n
1985–1989	431
1990–1994	2028
1995–1999	1666
2000–2004	1188
2005–2009	2240
2010–2014	5878
2015–2019	9848
2020–2024	17,328
2025	4747

ADR = adverse drug reaction, WHO = World Health Organization.

## 4. Discussion

This study provides an updated global descriptive overview of spontaneously reported ADRs associated with propofol using data from the WHO-VigiAccess database. By summarizing the distribution of ADRs according to MedDRA System Organ Class (SOC), patient demographics, geographical regions, and reporting trends over time, this study offers a comprehensive overview of the global pharmacovigilance profile of propofol. The findings demonstrate that propofol is associated with a broad spectrum of reported ADRs affecting multiple organ systems, with vascular, cutaneous, neurological, respiratory, immune, and cardiovascular disorders representing the most frequently reported categories.^[38-40]^ The predominance of cutaneous and vascular events aligns with known risks such as injection-site pain, thrombophlebitis, hypotension, and flushing.^[19,32,34]^ The frequency of general systemic and immune-related reactions supports recognition of hypersensitivity and anaphylactoid phenomena, which, though rare, can be fatal.

The presence of nervous system and respiratory disorders among the top categories reflects the drug’s pharmacodynamic action as a central nervous system depressant. These findings parallel clinical trial data showing high rates of bradycardia, hypoventilation, and loss of airway reflexes.^[4,41,42]^ Reports of metabolic, hepatic, and cardiac complications underscore ongoing concern regarding propofol infusion syndrome, particularly in critically ill patients receiving high-dose or prolonged infusions.^[43-45]^ A higher proportion of reports was observed among adults aged 45 to 64 years and male patients. Because VigiAccess lacks information on the total number of exposed patients and prescribing patterns, these findings should be interpreted as reporting distributions rather than indicators of differential risk.

While causality cannot be established in spontaneous databases, these descriptive findings complement controlled trial data, providing early warning signals of emerging trends. Regular updates of such analyses are critical for risk management, informing dosage guidelines, and enhancing education for clinicians handling long-term sedation.

## 5. Conclusion

This study provides an updated global descriptive overview of spontaneously reported ADRs associated with propofol using the WHO-VigiAccess database. The findings indicate that reported ADRs most frequently involve vascular, skin and subcutaneous tissue, general, nervous system, respiratory, immune, and cardiac disorders, highlighting the diverse clinical manifestations associated with propofol use. These results should be interpreted as reporting patterns rather than estimates of incidence or comparative risk because spontaneous reporting databases lack exposure denominators and do not permit causality assessment. Nevertheless, this study contributes valuable real-world pharmacovigilance evidence and emphasizes the importance of continued post-marketing surveillance, timely ADR reporting, and future analytical pharmacovigilance studies using individual case-level data to further characterize the safety profile of propofol, consistent with previous studies highlighting the complementary role of spontaneous reporting systems in detecting and monitoring drug safety signals.^[46,47]^

## 6. Limitations

This study has several limitations. As a spontaneous reporting database, WHO-VigiAccess is subject to under-reporting, reporting bias, duplicate reports, and incomplete clinical information. The public platform provides only aggregated data, preventing assessment of patient-level variables, concomitant medications, and other potential confounders. In addition, the absence of exposure denominators precludes estimation of incidence rates or comparative risks, and VigiAccess does not permit disproportionality analyses or comparisons with other IV anesthetic agents. Therefore, the findings should be interpreted as descriptive reporting patterns rather than evidence of causality or relative safety. Despite these limitations, this study provides a comprehensive global overview of reported propofol-associated ADRs and supports ongoing pharmacovigilance efforts.

## Acknowledgments

The authors extend their appreciation to Umm Al-Qura University, Saudi Arabia for funding this research work through grant number: 26UQU4420038GSSR08.

## Author contributions

**Conceptualization:** Nasser M. Alorfi, Ammar Aldabbagh.

**Data curation:** Nasser M. Alorfi, Waleed Saleh Alghamdi.

**Formal analysis:** Nasser M. Alorfi.

**Methodology:** Samah H.O. Zarroug.

**Resources:** Nasser M. Alorfi, Ahmed Abdullah Niyazi, Nasser M. Aldekhail.

**Software:** Nasser M. Alorfi, Abdulrhman M. Alansari.

**Supervision:** Nasser M. Alorfi, Nihal Abdalla Ibrahim.

**Validation:** Nasser M. Alorfi, Sarah S. Al Ghamdi, Mustfa Faisal Alkhanani, Tariq Omar Ali.

**Visualization:** Nasser M. Alorfi, Mansour M. Alourfi, Hana K Abdalla.

**Writing – original draft:** Nasser M. Alorfi, Faris A. Alsolami.

**Writing – review & editing:** Nasser M. Alorfi.
